# A smartphone intervention for adolescent obesity: study protocol for a randomised controlled non-inferiority trial

**DOI:** 10.1186/1745-6215-15-43

**Published:** 2014-01-31

**Authors:** Grace O’Malley, Mike Clarke, Amanda Burls, Sinéad Murphy, Nuala Murphy, Ivan J Perry

**Affiliations:** 1 Physiotherapy Department, Childhood Obesity Service, Temple Street Children’s University Hospital, Temple Street, Dublin 1, Ireland; 2 Department of Epidemiology and Public Health, University College Cork Western Gateway Building, Western Road, Cork, Ireland; 3 MRC Hub for Trials Methodology Research, Institute of Clinical Sciences, Block B, Queens University Belfast, Royal Vctoria Hospital, Belfast BT12 6BA, Ireland; 4 School of Health Sciences, Northampton Square, London EC1V 0HB, UK; 5 Obesity Service, Temple Street Children’s University Hospital, Temple Street, Dublin 1, Ireland; 6 Paediatric Endocrinology, Temple Street Children’s University Hospital, Temple Street, Dublin 1, Ireland; 7 Childhood Obesity Service, Physiotherapy Department, Temple Street Children’s University Hospital, Temple Street, Dublin 1, Ireland

**Keywords:** Obesity, Smartphone, Adolescent, Behavioural intervention, Mobile health, Telemedicine

## Abstract

**Background:**

There are few evidence-based mobile health solutions for treating adolescent obesity. The primary aim of this parallel non-inferiority trial is to assess the effectiveness of an experimental smartphone application in reducing obesity at 12 months, compared to the Temple Street W82GO Healthy Lifestyles intervention.

**Methods/design:**

The primary outcome measure is change in body mass index standardised deviation score at 12 months. The secondary aim is to compare the effect of treatment on secondary outcomes, including waist circumference, insulin sensitivity, quality of life, physical activity and psychosocial health. Adolescents with a body mass index at or above the 98th percentile (12 to 17 years) will be recruited from the Obesity clinic at Temple Street Children’s University Hospital in Dublin, Ireland. W82GO is a family-based lifestyle change intervention delivered in two phases over 12 months. In the current study, participants will be randomised for phase two of treatment to either usual care or care delivered via smartphone application. One hundred and thirty-four participants will be randomised between the two study arms. An intention-to-treat analysis will be used to compare treatment differences between the groups at 12 months.

**Discussion:**

The results of this study will be disseminated via open access publication and will provide important information for clinicians, patients and policy makers regarding the use of mobile health interventions in the management of adolescent obesity.

**Trial registration:**

Clinicaltrials.gov NCT01804855.

## Background

Childhood obesity in Ireland is a major problem: 19% of 9-year-old children are overweight and 7% are obese [1]. Child and adolescent obesity is associated with multiple physical and psychological co-morbidities [2] and, although effective treatments are available, there is considerable room for improvement. For example, a Cochrane Review reported sufficient evidence for the treatment effect of well-targeted interventions and its meta-analyses found a reduction in body mass index standardised deviation score (BMI SDS) of 0.14 at 12 months [3].

The Temple Street W82GO Healthy Lifestyles programme (W82GO) is a family-based multidisciplinary outpatient treatment for child and adolescent obesity (http://w82go.ie/). In a prospective study of the W82GO programme, significant reductions in BMI SDS have been described after 12 months of treatment [4]. However, although the beneficial effects of face-to-face family-based treatment have been described [5], it remains uncertain whether such treatments can be effective when delivered to potentially greater numbers using a mobile health approach.

Data exist regarding the use of technology [6] and mobile applications in a variety of clinical areas, including patient support [7], smoking cessation [8], diabetes management [9], and cardiac rehabilitation [10], but there is a dearth of data regarding the use of mobile health approaches in the management of adolescent obesity.

## Aim

The primary aim of this project is to assess the impact of a smartphone application compared with usual care on BMI SDS over 12 months in adolescents who are obese (12 to 17 years). Secondary outcome measures are waist circumference, insulin sensitivity, quality of life, physical activity and psychosocial health.

## Methods/design

### Trial design

Randomised trial of 12 months duration with parallel groups and assessor blinding.

### Participants

Children and adolescents attending Temple Street Children’s University Hospital in Dublin Ireland for obesity management will be recruited. Children are referred to the obesity clinic by their general practitioner, or primary healthcare team.

#### ***Eligibility criteria***

• Inclusion criteria: child aged between 12 and 17 years, child BMI >98th percentile, child fluent in English, parent(s) willing to participate in the programme with their child and completion of written informed consent and assent prior to any study-specific procedures.

• Exclusion criteria: severe intellectual difficulties which would limit the child’s ability to engage in group activity, obesity secondary to genetic condition, limitations to engaging in physical activity (for example, active musculoskeletal injury) or use of medication known to effect body weight, limitations to using a smartphone device and known family issues that would affect general compliance and attendance at follow-up visits.

### Recruitment

Children will be recruited from medical clinics and given written information regarding the study. Families wishing to participate in the study will be asked to complete parental consent and age-appropriate child assent forms. The motivation and expectations of both adolescents and parents will be evaluated and the alternative treatment options will be discussed (such as 1:1 treatment in outpatient clinic). It will be made clear to parents and their adolescents that by participating in the study they will have a 50:50 chance of being treated in a standard manner or with the smartphone intervention for phase 2 of treatment.

Based on our calculations, we will need to recruit 134 adolescents for randomisation over an enrolment period of 18 months.

This study will be conducted in agreement with the ‘Declaration of Helsinki’. The study has ethical approval from The Children’s University Hospital Ethics Board (11–033). All parents and adolescents will give their written informed consent. Modifications to this study protocol will be communicated to all those involved in the study: data monitoring committee, trial steering committee, clinical team, study sponsor, and study funders.

### Randomisation

Adolescents who are eligible will be randomised to either a W82GO (usual care) group or a smartphone experimental group by the research team using a secure online randomisation system with full allocation concealment. They will be stratified by gender and parental obesity, and 134 adolescents will be randomised in total using a 1:1 randomisation ratio. After randomisation, adolescents will receive a study code, which will be used to analyse all the data related to that child.

### Intervention

#### ***Assessment***

In keeping with routine practice, adolescents and their parents are assessed by the multidisciplinary team, which allows the team to build trust with the family. Assessment by the multidisciplinary team includes the following.

##### 

**Paediatrician** During this visit, general information is collected concerning pregnancy, birth, and early childhood development. The medical history of the family and the child is discussed along with any concerns that the child or parents have. The causal effects of obesity are discussed along with the medical complications that exist.

The physical examination investigates the presence of acanthosis nigricans, possible dysmorphic features, and hirsutism. Pubertal status is recorded according to Tanner and laboratory tests are requested.

##### 

**Dietitian** In this visit, the nutritional intake of the child is detailed in tandem with the eating behaviours of the family. The child is given a food diary to be completed over two weekend days and one weekday in order to provide an indication of micro- and macro-nutrient intake. The session is also used to introduce information regarding nutrition and healthy eating behaviour.

##### 

**Physiotherapist** The adolescent undergoes a physical examination to screen for musculoskeletal problems (for example, impaired gait or balance) and to measure physical activity levels, blood pressure, sedentary behaviour, sleep, cardiorespiratory fitness and quality of life.

##### 

**Psychologist** This session provides an opportunity for the family to discuss their motivations for becoming healthier. The role of the parent is highlighted and practical strategies to promote a positive environment at home are discussed. Issues such as body image, bullying and self esteem are assessed.

### Outcomes

#### ***Primary outcome***

##### 

**Anthropometric parameters** Body weight is measured to the nearest 0.1 kg using an electronic scale (SECA, Vogel & Halke, Hamburg, Germany) and height to the nearest 0.1 cm with a stadiometer (SECA, Vogel & Halke) in light clothing and without shoes. Measures are taken in triplicate and mean values calculated. Waist circumference is measured with an anthropometric tape midway between the lower rib margin and the iliac crest at the end of gentle expiration. BMI is calculated as weight/height squared (kg/m^2^). Subjects are classified as obese if they plot above the 98th percentile for BMI on the Irish growth charts. The primary outcome, BMI SDS, is calculated using UK reference data (Cole LMS method).

#### ***Secondary outcomes***

##### 

**Blood sample analysis** Blood samples are taken by venipuncture after an overnight fast by an experienced paediatric phlebotomist. Fasting samples are taken to measure glucose, insulin, total cholesterol, high density lipoprotein, low density lipoprotein, triglycerides, thyroid function, glycated haemoglobin, and the liver enzymes aspartate transaminase and alanine transaminase.

##### 

**Insulin resistance** The Homeostasis Assessment Model for insulin resistance formula [11] is used as an index of insulin resistance:

$$\left(\mathrm{fasting}\;\mathrm{insulin}\left(\mathrm{mU}/\mathrm{ml}\right)\times \mathrm{fasting}\;\mathrm{glucose}\left(\mathrm{mmol}/l\right)\right)/22.5$$

##### 

**Blood pressure** Blood pressure measurements (Omron, Kyoto, Japan) are taken in a relaxed sitting position, in triplicate. The last measurement is used for analyses [12].

##### 

**Physical activity** Objective measurement of physical activity will be completed using 7 days of accelerometry data (Geneactiv accelerometer, Activinsights, Kimbolton, UK). The Physical Activity Questionnaire for Adolescents will be used to measure subjective physical activity in adolescents. This is a 7-day self-report measure, which is reliable and valid for use in adolescents aged 7 to 19 years [13].

##### 

**Physical fitness** Cardiorespiratory fitness is measured with a submaximal treadmill test (modified Balke test) with heart rate, rate of perceived exertion and oxygen saturation monitoring [14]. The test takes between 10 and 15 minutes or until 85% of the maximum heart rate is achieved.

##### 

**Musculoskeletal screen** The physiotherapist screens the musculoskeletal system in order to identify any issues that might cause physical limitation to the child. The screening procedure has been detailed in full previously [15]. If there are musculoskeletal concerns identified, the symptoms will be treated as appropriate. Motor skill is evaluated using the balance and coordination subscale of the Bruinnincks Oteresky Test of Motor Proficiency (Pearson, TX, USA).

##### 

**Health-related quality of life** The Pediatric Quality of Life Inventory is used as a measure of health-related quality of life and has been used in obese cohorts previously [16].

##### 

**Psychosocial health** Social, behavioural and emotional functioning is assessed using the Child behaviour Checklist/Youth Self Report [17] and self concept by The Piers Harris Questionnaire [18].

### Treatments

W82GO is underpinned by behavioural change theory (transtheoretical model and social cognitive theory) and uses strategies including stimulus control, self-monitoring, positive reinforcement, goal setting and problem solving to facilitate lifestyle change. The intervention is described in full in Additional file 1. W82GO consists of phase 1 and phase 2 treatments.

#### ***Phase 1 treatment***

Phase 1 is the initial intensive phase and consists of six weekly sessions (0 to 6 weeks) for adolescents and their parents. These sessions last 2 hours and incorporate educational and practical sessions to increase physical activity, improve nutrition, increase sleep and reduce obesity.

#### ***Phase 2 treatment***

Phase 2 is a maintenance phase. Upon completion of phase 1, participants return with their parents for three 3-monthly booster maintenance sessions over 46 weeks [4]. These sessions are aimed to encourage the family to continue with lifestyle change and to manage barriers to change.

In the current study, having completed phase 1 of treatment, participants will be randomised for phase 2 to either usual care or smartphone care.

The smartphone application incorporates evidence-based behavioural change tools such as self-monitoring, goal setting, and peer support. Evidence-based tips are sent to the user in the form of a text tip, a video tip or an image tip. The tips aim to increase the knowledge of the participant with regard to healthy eating, physical activity, physical fitness and sleep. The user is encouraged to engage in daily goal setting to increase physical activity level and sleep, increase water intake, reduce intake of sugar and fat and to increase intake of fibre, fruits and vegetables. In addition, the user is encouraged to monitor their progress by reviewing their goals daily and by entering their height and weight measurements (which are charted over time).

**Figure 1:**
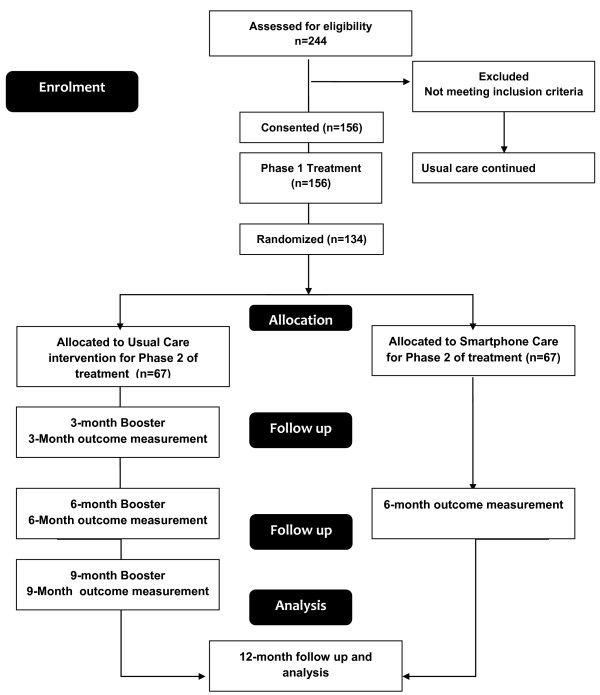
Study flow chart.

Those randomised to usual care will return for three booster sessions and for measurement of primary and secondary outcomes at 6 and 12 months. Those in the smartphone group will use the smarphone app and will return for measurement of outcomes at 6 to 12 months. Contact with the participants will be ensured throughout the study by monthly phone-calls from the principal investigator. See Figure 1.

### Power calculation

This study is a non-inferiority trial to determine whether a smartphone intervention could be used as a treatment in adolescent obesity management. The null hypothesis is that the smartphone intervention will have a positive effect on BMI SDS but that this change will be inferior to usual care. Based on a 0.21 reduction of BMI SDS at 12 months, a standard deviation of 0.24 in the usual care group and a non-inferiority limit of 0.12, the sample size required at 80% power will be 50 per group or 100 total. To allow for expected dropout the total number of adolescents to be recruited will be 134.

### Blinding

After randomisation and assignment to the study group, participants will be given a study identification number. The assessor of the primary outcome will not know the allocated intervention for the participant, to reduce the risk of bias. Given the nature of the intervention, it is not possible to blind the participants or the care providers. An independent data analyst will be blinded during the trial by the use of a fully anonymised dataset.

### Electronic health considerations

Given the dynamic nature of electronic health trials, there may be changes to the methodology after trial commencement and, if so, these will be detailed in full. Examples include major bug fixes in the software, changes in the functionality or content of the application and any unexpected events that might influence study outcomes (such as system failures or downtimes).

### Data collection and management

Primary and secondary outcome measurement will take place during initial baseline assessment, at 6 months and at 12 months. Participants who drop out of the trial will be invited to attend these follow-up visits for measurement. Data will be stored on password-protected firewalled computers, which are accessible only to members of our research team and those involved in managing the application software. Blood samples will be labelled with the patient’s initials and medical record number and stored in laboratories that are locked when not in use. With the permission of the participant (or parent/guardian), the blood samples and information collected during this research study may be stored indefinitely and used by our research group for future studies. Any information about individual participants derived from the additional studies will be kept confidential. The participant may at any time request that the blood samples be destroyed. When the results of the research are published or discussed in conferences, no information will be included that would reveal the identity of any participant unless their specific consent had been obtained. Data will be entered using double-data entry to assure quality control.

### Data analysis

All statistical analyses will be performed using SPSS (version 20, IBM, NY, USA). Descriptive statistics will be used to characterise demographic, clinical and laboratory variables. Distributions of primary and secondary outcomes will be examined using histograms and box plots for evidence of deviation from a normal distribution. If the distribution of a continuous outcome variable shows skewness or other marked departure from normality, mathematical transformations will be applied before using inferential techniques that require normality or alternative non-parametric methods (for example, bootstrap). An intention-to-treat analysis will be used to measure change in BMI SDS over time between the two groups and data will be adjusted for baseline age, BMI and gender.

### Data monitoring

A data monitoring committee will insure oversight of the trial and will meet with the principal investigator quarterly. This will provide independent review as to whether study participants are being exposed to unreasonable risks and will monitor study progress and integrity. The data monitoring committee will meet to review data from the ongoing trial (outcome data and data regarding adverse events) and make recommendations to the trial steering committee. Data will be managed in accordance with Temple Street Children’s University Hospital and UCC data protection policies. Any identifiable information that is obtained in connection with this study will remain confidential and will be disclosed only with the participant’s permission or as required by Irish or EU law.

### Adverse events

At each visit, participants and their parents will complete an adverse events form in order to capture any unintended effects of the trial. Spontaneous reporting of adverse events will be possible by calling the study phone line.

## Discussion

The results of the trial will be communicated to study participants, research collaborators, funders and the general public via a scientific publication and lay summary. The findings are expected to guide further development of a telemedicine system for the management of clinical obesity in adolescence. Ethical concerns specific to telemedical interventions have been considered such as the provision of web-enabled devices to minors, ensuring the security of patient data via online systems, and the use of devices in healthcare delivery which may expose a child to radio-frequency electromagnetic fields.

## Trial status

Recruiting.

## Abbreviations

BMI: body mass index; SDS: standardised deviation score; W82GO: Temple Street W82GO Healthy Lifestyles programme.

## Competing interests

The authors declare that they have no competing interests.

## Authors’ contributions

GOM, MC, SM and IJP are responsible for the design of the study and contributed to the intellectual content of the protocol. GOM and AB are responsible for the design and development of the smartphone app. GOM and SM are responsible for the implementation of the intervention, data collection, data analysis and drafted the study protocol with suggestions and contribution of all other authors. GOM, IP and NM obtained financial support. All authors read and approved the final manuscript.

## Supplementary Material

Additional file 1 Describes the full content of the evidence-based Temple Street W82GO Healthy Lifestyle Programme using a modified intervention mapping approach. Click here for file
