# Supplementary material for: A smartphone intervention for adolescent obesity: study protocol for a randomised controlled non-inferiority trial
Source: Trials. 2014 Jan 31;15:43. doi: 10.1186/1745-6215-15-43 (PMC3937237; doi:10.1186/1745-6215-15-43)
Supplement: Additional file 1 — Describes the full content of the evidence-based Temple Street W82GO Healthy Lifestyle Programme using a modified intervention mapping approach. [file 1745-6215-15-43-S1.doc]

**Additional File 1**

**Development of Temple Street w82go healthy lifestyles programme**

**introduction**

As childhood obesity in Ireland rises,[1] a slew of childhood health disorders are presenting to our healthcare facilities. The negative impact of obesity on the developing child’s health includes respiratory, cardiovascular, musculoskeletal, and metabolic effects.[2] The development of pediatric obesity is associated with a number of environmental, genetic, and medical conditions.[3,4] However, the vast majority of childhood obesity is exogenous in nature and is more appropriately conceptualized as a ‘lifestyle disease’ with a variety of environmental determinants.[5]

**objective**

In response to the serious issue of childhood obesity, the Temple Street W82GO Healthy Lifestyles Treatment Service (W82GO) was developed. The following describes how the service was designed based on an intervention mapping approach.[6]

**INTERVENTIONS**

**Needs Assessment**

*Identifying the problem*

In Ireland, up to 19% of children are overweight and up to 7% are obese.[7] Increasing childhood obesity has in turn increased the number of children presenting with associated co-morbidities.[8-12] Obesity related co-morbidities represent a serious challenge to the child, the family, the healthcare system, and the community at large. As a child who is obese has a high probability of becoming an obese adult, intervention should begin in childhood.

*Identifying a solution*
There is sufficient evidence to justify well-targeted treatment of children with obesity[13] and meta-analyses indicate that treatment interventions can produce significant and clinically meaningful reductions in obesity.[13] Positive persistent results may be obtained with treatment programmes which are family-based and combine cognitive-behavioral, nutrition and physical activity strategies in a lifestyle centered approach .[14] Prior the the development of W82GO, no such treatment option was available in Irish primary or secondary care settings. W82GO is a multidisciplinary service with input from a pediatrician; a dietitian; a physiotherapist, a nurse and a pediatric clinical psychologist. Children who have a Body Mass Index (BMI) ≥98^th^ percentile can be referred for treatment. The W82GO service has been modeled on best practice recommendations,[14] and the primary intervention goal is a reduction in BMI SDS.

**Formulation of change objectives**

In keeping with best practice evidence, the overall aim of the intervention is to enable permanent change in a child’s eating habits and level of physical activity.[14] Thus, the focus of treatment is not only on reducing obesity, but also rather on the behavioral and attitudinal changes needed to establish and maintain a healthy lifestyle. Such lifestyle changes are determined by a number of performance objectives (e.g. such as a reduction of excessive consumption of energy dense foods).

The behaviors and attitudes of the family in relation to health promotion are identified during a thorough assessment and specific tailored change objectives are developed (Table 1). In order to achieve change in these variables a number of sub-behaviors are promoted such as self monitoring by the child and parent/s, self management, goal setting and problem solving. At every opportunity the service aims to empower the family to recognise behaviors that could be changed and encourages development of the skills required to make and sustain such changes.

Table 1

| ***Behaviour and attitudinal change*** |
| --- |
| *Behavioural Change Objectives* |
| Increase knowledge and understanding of the benefits of a healthy lifestyle |
| Encourage familial, parental and child attitudes and motivation around change |
| Promote health literacy (e.g. beliefs around behaviour and associated health |
| Improve eating behaviour of the family and child |
| Increase level of physical activity (within school and at home with family and friends) |
| Facilitate personal skills (e.g. self care and cooking skills) |
| Promote the physical and emotional health of the child |
| Improve general parenting skills |
| Increase problem solving skills |
| Improve communication within the family |
| Raise children’s awareness of and ability to deal with teasing and bullying |
| Increase children’s self esteem |
| Promote quality of life |
| Optimize the sleep routine of family |

**Methods and practical strategies to reach objectives**

Initial Referral

W82GO is grounded in behavioral change theory (transtheoretical model and social cognitive theory). The transtheoretical model is incorporated from the initial contact, when the service is described and the family’s level of interest is assessed. In line with other areas of obesity treatment,[15] the rate of non-attendance at initial appointment is high (25%) which may indicate the proportion of families at a less active phase of the change process.[16] Attempts are made at this point to increase engagement and move families along the cycle of change by providing both verbal and written information on the service.

Holistic Assessment
An initial screening appointment provides an opportunity to get to know the family, build trust, and screen for underlying medical conditions. Assessment by the MDT provides an opportunity to determine health literacy, health-beliefs and the physical and environmental variables that may act as barriers to change. Outcome measures taken during assessment are described in Table 2.

Participatory Development
The service has evolved based on strategies identified as effective in the literature[13] and incorporating feedback from parents and children attending the service. Participants are referred mainly from the local hospital catchment area, which is described as an area of high-to extreme disadvantage and deprivation.[17] As such the intervention has been developed to be culturally and socioeconomically sensitive. In order to facilitate differing family situations and dynamics the service offers both individual and group-based treatment.

Promoting Change via the social network

Based on social cognitive theory W82GO makes use of social influence to encourage lifestyle change via a supportive network of clinicians, family, and friends. The impact of the social network is maximized, by establishing the change process, as a social norm within the group. This is done through weekly goal review, whereby each family reports the goals they have worked on for the previous week and receives encouragement and praise from facilitators and fellow group members. During the games sessions the group is facilitated to work together and to encourage each other. In addition, during practical sessions observational learning is promoted such that families observe one-another’s changes and methods for managing problems that may arise (e.g. how parents manage adolescents resistant to changes within the home).

Family Involvement

Lifestyle change can be usefully understood as an active process involving the entire family. Parents are vital in reducing negative stimuli and increasing positive stimuli within the home and encouraging and reinforcing positive behaviors. Evidence suggests that family-based programmes, where parents take primary responsibility and act as agents for change are likely to be successful for managing childhood obesity.[18] Parental involvement in W82GO ensures that parents can function as models of positive lifestyle change and can reinforce positive eating and activity behavior through the use of rewards. Parents are encouraged to think creatively about their use of rewards and to identify non-monetary, non-food rewards (e.g. quality family time such as a family outing to a park). Family dynamics can also play an important role, as dual households (where parents are divorced or grandparents play a significant carer role) can lead to a lack of consistency in the support of healthy choices.

In addition to parental behaviors that relate directly to eating and physical activity, the service incorporates more general family interventions such as parenting skills and strategies to improve family communication. Evidence suggests that pediatric obesity programmes incorporating a broader family focus are successful.[19]

Self-efficacy Self-efficacy is promoted through the successful achievement of small goals at each stage of the intervention. Self-efficacy is promoted in practical exercise based and nutrition-related sessions, such as trips to the supermarket to develop participant’s skills in making healthy food choices. Situation specific self-efficacy is also promoted e.g. helping parents plan how to limit access to treats during festive occasions.

Table 2

| ***Assessment*** | |
| --- | --- |
| *Physical Measures* | |
| Anthropometric variables | Height (cm), Weight (Kg), Waist Circumference |
| Obesity Classifications | BMI, BMI percentile and BMI SDS |
| Laboratory Tests | Fasting glucose, insulin, cholesterol (total, HDL and LDL); triglycerides; AST; ALT; HbA1c) |
| Cardiovascular variables | Blood Pressure, Heart rate, Oxygen Saturations, |
| Cardiorespiratory fitness | 6MWT and/or Modified Balk Treadmill Test with  heart rate monitoring and rating of perceived exhaustion |
| Musculoskeletal Screen | Range of motion, pain, bony alignment, posture,  flexibility, strength, gait and balance. |
| *Lifestyle Measures* |  |
| Physical Activity Level: | Accelerometry (RT3) and questionnaire (MAQA/PAQ-C) |
| Quality of Life Level: | PedsQL |
| Dietary | Assessment and measures |
| Sleep | Sleep Disturbances Scale for Children |
| *Psychosocial Measures* |  |
| Social, Behavioural and Emotional functioning | Child Behaviour Checklist |
| Self concept: | The Piers Harris Questionnaire |
| Eating Habits: | Dutch Eating Behaviour Questionnaire. |

Communication

From point of referral to point of discharge the service aims to utilise optimal communication techniques. All service materials are written in clear and age-appropriate language and where possible a variety of media are used during education and practical sessions to reinforce learning. Throughout contact with the families motivational interviewing techniques are employed in order to facilitate a supportive atmosphere.[20]

Goal Setting

During the screening stage, families are encouraged to identify appropriate long-term objectives and to translate these into specific and achievable short-term goals. This helps to prepare the family for change based on their individual needs and wants. While engaged in treatment, the families are encouraged to continue with regular goal setting (e.g. through weekly goal review for families attending the group intervention). W82GO incorporates specific training in goal setting, managing barriers to change, and relapse prevention. Goals are based around the positive health behaviors that are promoted in the education and practical sessions and parents are taught skills needed to implement goals (e.g. steps needed to establish a healthy sleep routine).

**Programme development**

# Treatment Components

*Physiotherapy*: Low levels of physical activity during childhood have been associated with morbidity and mortality in adulthood.[21] The physiotherapy component of the service focuses on accurate physical assessment (Table 2). Physical activity can benefit children who are obese by increasing their lean body mass, increasing energy expenditure, and improving their metabolic and psychological profiles.[22] Independent of any effect on weight, the above changes justify the promotion of physical activity in children. Physiotherapy assessment evaluates global fitness in order to identify structural impairments which may limit time spent in the activity required for weight maintenance.[8] In addition, the barriers to physical activity are discussed and can assist with group goal setting around common obstacles. Where physical impairments are identified, a treatment plan is agreed. Treatment incorporates supervised exercise sessions which aim to establish movement and exercise as an enjoyable and rewarding experience for the children involved. Exercise sessions are fun, are tailored to the ability and preferences of the participants and aim to preserve lean mass; increase aerobic activity; and improve motor skill in a safe and secure environment. Exercise recommendations are specific to the individual child and incorporate both weight bearing and non-weight bearing activity depending on severity of obesity. Successful physical training becomes an important component in the process of enhancing the self-esteem of participants.[23] During the programme the attitudes of the child’s parents towards physical activity are also addressed and all members of the family are encouraged to partake in physical activity together (e.g. family outings).

In addition to the promotion of physical activity, sedentary pursuits are also discouraged. Television viewing and screen time have been directly related to the degree of obesity in childhood.[24] Similarly, given the impact of sleep on child health,[25] W82GO manages sleep difficulties as recommended.[26,27]

*Nutrition Component*: The increasing prevalence of obesity is likely related to changes in nutritional intake and decreasing children’s energy intake may be sufficient to reduce obesity.[28] W82GO aims to educate families about healthy eating and balancing energy intake with expenditure in the pursuit of optimal health and growth. Educational and practical sessions aim to increase the family’s awareness of healthy eating, appropriate portion sizes and swapping high energy-dense foods for more healthy and nutritious options. A main focus is on limiting energy-dense foods with low nutrient value such as sugary drinks.[29] Similarly, increased intake of fibrous foods which offer greater satiety is encouraged,[30] and excessive fat intake may contribute to weight gain and is discouraged.[31]

*Eating behavior*: The National Children’s food survey[32] indicated that 89% of all meals and snacks eaten by Irish school-children are eaten at home. As energy intake from restaurants and fast food outlets can be high,[33] the home environment has a critical influence on the dietary behavior of children. Parents play a direct role in children’s eating patterns through their behaviors, attitudes and feeding styles and W82GO encourages parents and children to improve their culinary skills in order to prepare healthy meals. In addition, the service promotes behaviors such as eating together at the table, the preparation of one family meal (rather than a variety of meals for each family member); regular meal-times; the avoidance of using food as a reward for good behavior; adequate chewing of each mouthful of food; and the promotion of adequate hydration to avoid excessive thirst in children (which may be interpreted as hunger). Similarly, the family learns to prepare meals of recommended portion sizes and learn techniques to avoid the over-consumption of energy-dense nutrient-low foods. Finally, W82GO assesses and provides support for children with binge-eating disorders.

*Psychological Component:* Although education on diet and activity are the core components of obesity treatment, educating children about these two aspects of healthy lifestyle is rarely sufficient to produce the necessary behavior change. In order to enhance behavior change, strategies and techniques from the field of behavioral psychology and cognitive behavioral therapy have been applied to the area of obesity.[34] W82GO incorporates specific behavioral and cognitive behavioral strategies in order to facilitate behavior change among participating families. These strategies include: stimulus control, self-monitoring, reinforcement, modeling, self-instructional training, and problem solving. For example, structured problem-solving strategies are encouraged whereby parents identify barriers to healthy behaviors and support each other in identifying a range of solutions to these barriers.

W82GO also aims to address the psychosocial issues that are associated with obesity (higher rates of psychological difficulties, in particular social difficulties, and lower self-esteem.[35,36] W82GO aims to enhance children’s resilience, psychological health, and self-esteem through direct input (information sessions and practical activities for participants to enhance their self esteem and teach appropriate strategies for managing teasing and bullying),[37-39] as well as indirect intervention, such as teaching strategies to enhance family communication and parenting skills.

# Treatment Phases

Following assessment families are invited to attend the intensive phase of treatment of W82GO (run two-hourly over 6-weeks for those in group sessions) and thereafter are invited to attend three three-monthly booster sessions in order to maintain contact and reinforce triggers for positive lifestyle changes. Children are discharged from the service when they maintain a BMI at or below the 95^th^ percentile for more than three months.

**Adoption, implementation, and evaluation**

The adoption and implementation of W82GO has been informed by stakeholder feedback and evaluation. Based on the response of families to a pilot programme the service has evolved to meet the needs and expectations of the majority of families referred. Programme materials such as educational materials, work packs, and a website ([www.w82go.ie](http://www.w82go.ie/)) have been developed and are used throughout the service. Results of the clinical effectiveness of the intervention are described in subsequent chapters.

# conclusion

# W82GO is an evidence-based pediatric obesity service delivered within an outpatient setting. Though the bulk of pediatric obesity prevention and treatment is most suited to a community environment the high prevalence of co-morbidities in the cohort indicate that hospital-based management is necessary and can be effective. Moreover, the Temple Street W82GO Healthy Lifestyles is designed so that it can be easily adapted for use in the community, so that it can benefit a wider population of children who are obese.

**Reference LisT**

1. Perry IJ, Whelton H, Harrington J, *et al.* The heights and weights of Irish children from the post-war era to the Celtic tiger. J Epidemiol Community Health 2009;63(**3**):262-4. Doi: 10.1136/jech.2008.079236
2. Ebbeling CB, Pawlak DB, Ludwig DS. Childhood obesity: public-health crisis, common sense cure. Lancet 2002;360(**9331**):473-82. Doi: 10.1016/S0140-6736(02)09678-2
3. Thorleifsson G, Walters GB, Gudbjartsson DF, *et al.* Genome-wide association yields new sequence variants at seven loci that associate with measures of obesity. Nat Genet 2009;41(**1**):18-24. Doi: 10.1038/ng.274
4. Wardle J, Carnell S, Haworth CM, *et al.* Evidence for a strong genetic influence on childhood adiposity despite the force of the obesogenic environment. Am J Clin Nutr 2008;87(**2**):398-404. Doi: 87/2/398
5. Hawkins SS, Cole TJ, Law C. An ecological systems approach to examining risk factors for early childhood overweight: findings from the UK Millennium Cohort Study. J Epidemiol Community Health 2009;63(**2**):147-55. Doi: 10.1136/jech.2008.077917
6. Bartholomew LK, Mullen PD. Five roles for using theory and evidence in the design and testing of behavior change interventions. J Public Health Dent 2011;71 Suppl 1:S20-33.
7. Layte R, McCrory C. Overweight and obesity among 9-year-olds. Dublin: Government Publications, 2011.
8. O'Malley G, Hussey J, Roche E. A pilot study to profile the lower limb musculoskeletal health in children with obesity. Pediatr Phys Ther 2012;24(**3**):292-8. Doi: 10.1097/PEP.0b013e31825c14f8
9. O'Malley G, Santoro N, Northrup V, *et al.* High normal fasting glucose level in obese youth: a marker for insulin resistance and beta cell dysregulation. Diabetologia 2010;53(**6**):1199-209. Doi: 10.1007/s00125-010-1693-0
10. Bell LM, Curran JA, Byrne S, *et al.* High incidence of obesity co-morbidities in young children: A cross-sectional study. J Paediatr Child Health 2011;47(**12**):911-7. Doi: 10.1111/j.1440-1754.2011.02102.x
11. Tounian P, Aggoun Y, Dubern B, *et al.* Presence of increased stiffness of the common carotid artery and endothelial dysfunction in severely obese children: a prospective study. Lancet 2001;358(**9291**):1400-4. Doi: 10.1016/S0140-6736(01)06525-4
12. Decaluwe V, Braet C, Moens E, *et al.* The association of parental characteristics and psychological problems in obese youngsters. Int J Obes (Lond) 2006;30(**12**):1766-74. Doi: 10.1038/sj.ijo.0803336
13. Oude Luttikhuis H, Baur L, Jansen H, *et al.* Interventions for treating obesity in children. Cochrane Database Syst Rev 2009(**1**):CD001872. Doi: 10.1002/14651858.CD001872.pub2
14. Thompson J. Management of obesity in Scotland: development of the latest evidence-based recommendations. Proc Nutr Soc 2010;69(**2**):195-8. Doi: 10.1017/S0029665110000066
15. Skelton JA, Beech BM. Attrition in paediatric weight management: a review of the literature and new directions. Obes Rev 2011;12(**5**):e273-81. Doi: 10.1111/j.1467-789X.2010.00803.x
16. Prochaska JO, Velicer WF. The transtheoretical model of health behavior change. Am J Health Promot 1997;12(**1**):38-48.
17. Small Area Health Research Unit. The National Deprivation Index For Health and Health Services Research, *SAHRU Technical Report,* 2007, December.
18. Young KM, Northern JJ, Lister KM, *et al.* A meta-analysis of family-behavioral weight-loss treatments for children. Clin Psychol Rev 2007;27(**2**):240-9. Doi: 10.1016/j.cpr.2006.08.003
19. Kitzmann KM, Beech BM. Family-based interventions for pediatric obesity: methodological and conceptual challenges from family psychology. J Fam Psychol 2006;20(**2**):175-89. Doi: 10.1037/0893-3200.20.2.175
20. Resnicow K, Davis R, Rollnick S. Motivational interviewing for pediatric obesity: Conceptual issues and evidence review. J Am Diet Assoc 2006;106(**12**):2024-33. Doi: 10.1016/j.jada.2006.09.015
21. Paffenbarger RS, Jr., Hyde RT, Hsieh CC, *et al.* Physical activity, other life-style patterns, cardiovascular disease and longevity. Acta Med Scand Suppl 1986;711:85-91.
22. Goldfield GS, Henderson K, Buchholz A, *et al.* Physical activity and psychological adjustment in adolescents. J Phys Act Health 2011;8(**2**):157-63.
23. Saelens BE, Epstein LH. Behavioral engineering of activity choice in obese children. Int J Obes Relat Metab Disord 1998;22(**3**):275-7.
24. Wiecha JL, Peterson KE, Ludwig DS, *et al.* When children eat what they watch: impact of television viewing on dietary intake in youth. Arch Pediatr Adolesc Med 2006;160(**4**):436-42. Doi: 10.1001/archpedi.160.4.436
25. Kong AP, Wing YK, Choi KC, *et al.* Associations of sleep duration with obesity and serum lipid profile in children and adolescents. Sleep Med 2011;12(**7**):659-65. Doi: 10.1016/j.sleep.2010.12.015
26. Jiang F, Zhu S, Yan C, *et al.* Sleep and obesity in preschool children. J Pediatr 2009;154(**6**):814-8. Doi: 10.1016/j.jpeds.2008.12.043
27. Iglowstein I, Jenni OG, Molinari L, *et al.* Sleep duration from infancy to adolescence: reference values and generational trends. Pediatrics 2003;111(**2**):302-7.
28. Swinburn B, Sacks G, Ravussin E. Increased food energy supply is more than sufficient to explain the US epidemic of obesity. Am J Clin Nutr 2009;90(**6**):1453-6. Doi: 10.3945/ajcn.2009.28595
29. Ludwig DS, Peterson KE, Gortmaker SL. Relation between consumption of sugar-sweetened drinks and childhood obesity: a prospective, observational analysis. Lancet 2001;357(**9255**):505-8. Doi: 10.1016/S0140-6736(00)04041-1
30. Johnson L, Mander AP, Jones LR, *et al.* Energy-dense, low-fiber, high-fat dietary pattern is associated with increased fatness in childhood. Am J Clin Nutr 2008;87(**4**):846-54. Doi: 87/4/846 [pii]
31. Jequier E. Is fat intake a risk factor for fat gain in children? J Clin Endocrinol Metab 2001;86(**3**):980-3.
32. IUNA. The National Children’s Food Survey. *Irish Universities Nutrition Alliance* 2005.
33. Bowman SA, Gortmaker SL, Ebbeling CB, *et al.* Effects of fast-food consumption on energy intake and diet quality among children in a national household survey. Pediatrics 2004;113(**1 Pt 1**):112-8.
34. Jelalian E, Saelens BE. Empirically supported treatments in pediatric psychology: pediatric obesity. J Pediatr Psychol 1999;24(**3**):223-48.
35. Epstein LH, Myers MD, Anderson K. The association of maternal psychopathology and family socioeconomic status with psychological problems in obese children. Obes Res 1996;4(**1**):65-74.
36. Mills JK, Andrianopoulos GD. The relationship between childhood onset obesity and psychopathology in adulthood. J Psychol 1993;127(**5**):547-51. Doi: 10.1080/00223980.1993.9914892
37. Puhl RM, Heuer CA. The stigma of obesity: a review and update. Obesity (Silver Spring) 2009;17(**5**):941-64. Doi: 10.1038/oby.2008.636
38. Bell SK, Morgan SB. Children's attitudes and behavioral intentions toward a peer presented as obese: does a medical explanation for the obesity make a difference? J Pediatr Psychol 2000;25(**3**):137-45.
39. Goldfield GS, Moore C, Henderson K, *et al.* Body dissatisfaction, dietary restraint, depression, and weight status in adolescents. J Sch Health 2010;80(**4**):186-92. Doi: 10.1111/j.1746-1561.2009.00485.x
40. O’Malley G, Brinkley A, Moroney K, McInerney M, Murphy S, Kileen S, Murphy N. Is the Temple Street W82Go Healthy Lifestyles Programme effective in reducing BMI SDS? Obesity Facts*,* 2012;5(**S10**):223. Doi: DOI:10.1159/000207438
